# Validation of an LC-MS/MS method for the quantitation of phytosterols derived from Aloe vera gel

**DOI:** 10.1016/j.mex.2022.101642

**Published:** 2022-02-24

**Authors:** Kazumi Nabeshima, Atsushi Mizutani, Eriko Misawa, Miyuki Tanaka, Koji Yamauchi, Fumiaki Abe

**Affiliations:** a Food Ingredients & Technology Institute, R&D Division, Morinaga Milk Industry Co., Ltd.; b Quality Assurance Division Quality Control Department, Morinaga Milk Industry Co., Ltd.

**Keywords:** Aloe vera gel, Phytosterol, Supercritical carbon dioxide extract, LC-MS/MS, Functional food

## Abstract

A method to quantitate five minor phytosterols named *Aloe* sterols identified from *Aloe vera* gel was validated using AVGP (*Aloe vera* gel powder) as the sample. To measure the *Aloe* sterols content, AVGP was extracted with chloroform/methanol (2:1, v/v) and analyzed by liquid chromatography-tandem mass spectrometry. The calibration curve revealed a high coefficient of determination (>0.999). The limit of quantification was 2.3–4.1 ng/mL. Average recoveries ranged from 95 to 105%. The intra-day and inter-day precision were 2.6–6.4% and 3.8–7.3%, respectively, confirming good method precision. *Aloe* sterols were also quantified in AVGE (*Aloe vera* gel extract) using this method. We showed that the composition ratio of each *Aloe* sterol in AVGP did not change in AVGE. Additionally, we measured the concentration of *Aloe* sterols in the capsule containing AVGE, and confirmed that it was stable even after 1 year of storage.

In conclusion, a quantification method was established to simultaneously measure multiple plant sterols with similar structures.

• A quantification method to simultaneously measure several plant sterols with similar structures was established.

• Results from the intra-day precision and the inter-day precision confirmed good precision.

• This method can be applied to processed raw materials and/or foods in long-term storage.

## Graphical abstract

**Figure:**
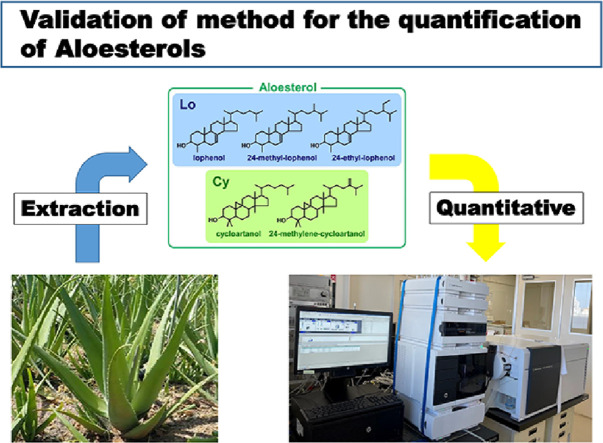

**Specifications table**

**Table:** 

Subject area;	Agricultural and Biological Sciences
More specific subject area;	Analytical Chemistry
Method name;	A liquid chromatography-tandem mass spectrometry method
Name and reference of original method;	N.A.
Resource availability;	N.A.

## Introduction

*Aloe barbadensis* Miller (*Aloe vera*), a tropical plant belonging to the family *Liliaceae*, contains various pharmacologically active ingredients [1]. In our previous study, we attempted to isolate hypoglycemic ingredients from *Aloe vera* gel and succeeded in the identification of five minor plant sterols (lophenol, 24-methyl-lophenol, 24-ethyl-lophenol, cycloartanol, and 24-methylene-cycloartanol), named *Aloe* sterol [2].

We performed several clinical studies, and reported that *Aloe* sterol could improve liver function [3,4], blood sugar levels [5], and skin function [6,7,8]. Patient safety was confirmed in the clinical trials using *Aloe* sterol capsules (cycloartanol 140.0 μg/day, lophenol 117.6 μg/day) as a test food [4]. Therefore, it was suggested that *Aloe* sterol can be used as an ingredient in functional foods.

To develop functional foods, a method capable of accurately and quickly quantifying functional ingredients is required. The most common technique used for the analysis of phytosterols is gas chromatography (GC) [9] with a flame ionization detector or mass spectrometer. To analyze *Aloe* sterols via GC, it is necessary to extract them with an organic solvent, then saponify to remove the matrix, and derivatize the compounds with trimethylsilyl ether [10. Analysis by liquid chromatography-tandem mass spectrometry (LC-MS/MS), on the contrary, does not require derivatization and saponification [10,11]. Additionally, LC-MS/MS enables the quantification of each of the five components in *Aloe* sterol. Thus, it is considered that LC-MS/MS analysis can make the measurements more efficient.

The purpose of this study was to develop a sensitive and accurate method for quantifying *Aloe* sterol using LC-MS/MS. We validated the developed method (evaluation of linearity, calculation of the limit of quantification and relative standard deviation (RSD), recovery test, and precision check) to confirm good method precision.

Using the developed method, we measured the content of *Aloe* sterol in the extracts of AVGP (*Aloe vera* gel powder) and AVGE (*Aloe vera* gel extract), obtained by supercritical carbon dioxide (CO_2_) extraction. We also investigated the effect of supercritical CO_2_ extraction on the composition of each component of *Aloe* sterol in *Aloe vera* gel. Furthermore, we examined the effect of storage on the *Aloe* sterol concentration in capsules containing AVGE.

*Method details

## Materials and methods

### Reagents and chemicals

**Fig. 1:**
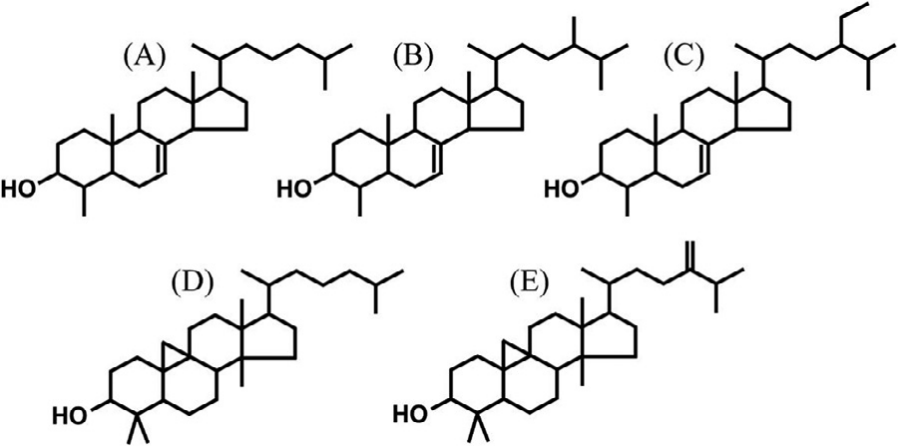
Structures of (A) lophenol, (B)24-methyl-lophenol, (C)24-ethyl-lophenol, (D)cycloartanol and (E)24-methylene-cycloartanol.

Figure 1 shows the structures of lophenol, 24-methyl-lophenol, 24-ethyl-lophenol, cycloartanol, and 24-methylene-cycloartanol. Standards of these components (>98% purity) were purchased from KNC Laboratories Co., Ltd. (Hyogo, Japan). Guaranteed-grade chloroform and methanol were obtained from FUJIFILM Wako Pure Chemical Corporation (Tokyo, Japan) and used for standard solution and sample preparation.

LC/MS-grade methanol, formic acid, and acetonitrile were purchased from FUJIFILM Wako Pure Chemical Corporation and used for the mobile phase.

Preparation of calibration standard

The stock solution was prepared by dissolving 1 mg of each *Aloe* sterol in 1 mL methanol/chloroform (1:4, v/v), and was stored at -18 °C.

A working solution containing all five standards was prepared at a concentration of 1 μg/mL by pipetting the required volume of the individual stock solution and diluting with methanol. The solutions of calibration standard (10, 20, 40, 80, and 160 ng/mL) were prepared by diluting the working solution with methanol.

### Method validation

Linearity

The solutions of calibration standard (10, 20, 40, 80, and 160 ng/mL) were used to build the calibration curve and its coefficient of determination was calculated.

Calculation of the limit of quantification and relative standard deviation (RSD)

The calibration standard (10 ng/mL) was analyzed five times to calculate the standard deviation, limit of quantification (10 S/N) [12], and RSD.

Recovery test

A recovery test was performed to confirm the accuracy of this method. Recovery was calculated as follows:

Recovery (%) = (C spiked – C sample) / C standard × 100

C spiked: concentration of the sample to be spiked

C sample: concentration of the sample

C standard: concentration of the standard

Precision

The method precision was assessed by repeatability and intermediate precision. The repeatability was calculated by measuring the same sample 5 times on the same day. The intermediate precision was determined by the results of samples (*n* = 2 /day) prepared and analyzed on six different days. The RSDs were calculated.

### LC-MS/MS analysis

Liquid chromatography was performed using the Agilent Technology 1260 system equipped with a degasser, binary pump, multisampler, and column compartment. Chromatographic separation was performed on an Ascentis Express C18 HPLC column (2.1  ×  100 mm, 2.7 μm particles; Sigma-Aldrich Supelco) with a guard column (2.1  ×  5 mm, 2.7 μm particles; Sigma-Aldrich Supelco). The analytic conditions were as follows: injection volume, 10 μL; flow rate, 0.4 mL/min; column temperature, 50 °C; multisampler, 10 °C. Solvent A was acetonitrile/water (90:10 v/v) and solvent B was methanol/water/formic acid (900:100:1, v/v/v). The gradient program was as follows: solvent A 100%, solvent B 0% (0–8.5 min), solvent A 0%, solvent B 100% (8.5–24 min), and solvent A 100%, solvent B 0% (24–36 min).

**Fig. 2:**
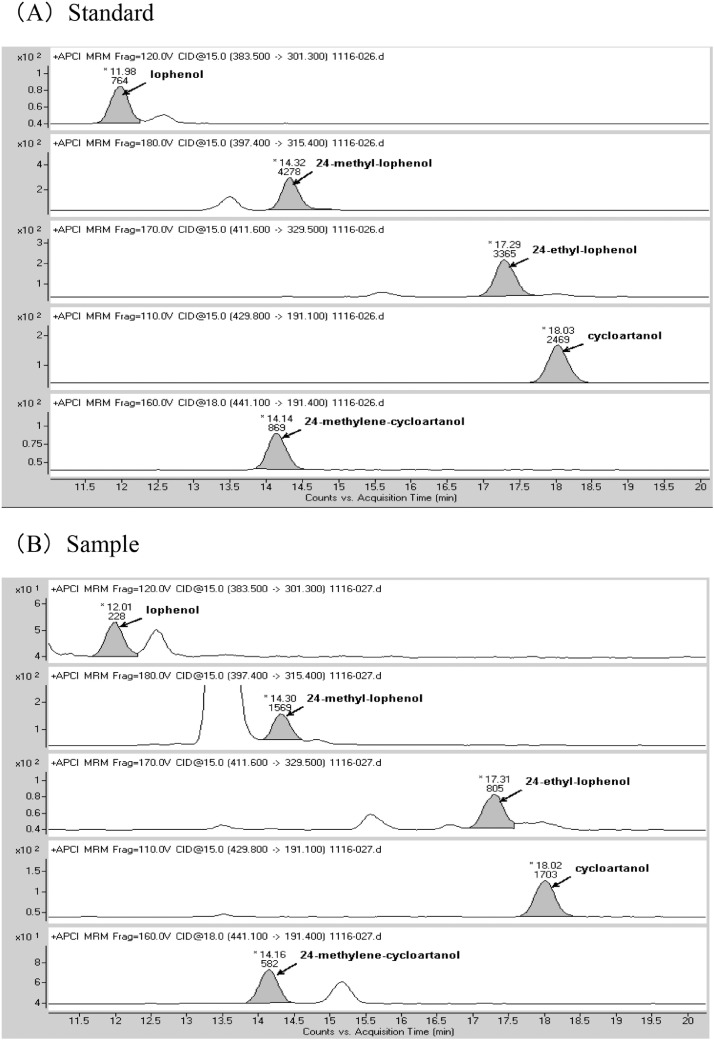
Chromatograms of (A) 160 ng/mL standard solution, (B) measurement sample.

For MS/MS, we used Agilent Technology 6460 Triple Quad LC/MS. The parameters are as follows: nebulizer pressure, 20 psi; drying gas flow rate, 4 mL/min; temperature, 325 °C; capillary voltage, 4,500 V; collision gas, N_2_. The other parameters are shown in Table 1. Chromatograms of the standard solution and a measurement sample are shown in Fig. 2.

**Table 1:** MS/MS parameters of *Aloe* sterols.

Compound	precursor ion(m/z)	product ion (m/z)	fragmentor voltage (V)	Collision energy(V)	polarity
lophenol	383.5	301.3	120	15	+
24-methyl-lophenol	397.4	315.4	180	15	+
24-ethyl-lophenol	411.6	411.6	170	15	+
cycloartanol	429.8	191.1	110	15	+
24-methylene-cycloartanol	441.1	191.4	160	18	+

### Sample preparation

AVGP

AVGP, a fine powder from dried and ground *Aloe vera* mesophyll, was used as a sample for the validation of the developed quantitative method. For the sample preparation, AVGP (1 g) was added to a 50 mL flask, followed by 40 mL of the extraction solvent (chloroform/methanol (2:1, v/v)). This mixture was dissolved by sonication for 10 min at 25 °C. The volume was then adjusted to 50 mL with the extraction solvent. The sample suspension (5 mL) was centrifuged (1150 × *g*, 25 °C, 5 min) and the supernatant (2 mL) was collected. The solvent was eliminated by evaporation at 40 °C under a nitrogen stream. The dried residue was dissolved in 20 mL of methanol in ambience by sonication for 15 min and filtered through a 0.22 μm filter (PVDF, Tomsic, Japan) to obtain a measurement sample.

### AVGE oil

AVGE is an extract containing a hydrophobic component, obtained from the dried *Aloe vera* mesophyll by extraction using supercritical CO_2_ as the solvent. The manufacturing process conditions are based on our previous research [13]. AVGE was dispersed in an edible oil to prepare AVGE oil, for use as food materials. Since the AVGE oil has a high viscosity, it was first warmed in a 50 °C water bath and mixed well to reduce the viscosity to prepare for measurement. The sample (0.5 g) was then added to a 50 mL flask, followed by 40 mL of the extraction solvent. This mixture was dissolved by sonication for 10 min at room temperature, and its volume adjusted to 50 mL with the extraction solvent. The sample suspension (2 mL) was added to a 25 mL flask, followed by 25 mL of the extraction solvent. The solvent was removed by evaporating 2 mL of this solution under a nitrogen stream at 40 °C. The dried residue was dissolved in 4 mL of methanol at room temperature by sonication for 10 min, after which 1 mL of distilled water was added, mixed well, then filtered through a 0.22 μm filter.

The filtered mixture was pretreated using a Solid Phase Extraction (SPE) cartridge (Oasis HLB 6cc (200 mg), Waters, MA, USA) to clean the matrix. The SPE cartridge was conditioned with 5 mL of methanol/water (80:20, v/v) and then flowed with the filtered extraction solution. Subsequently, the cartridge was washed with 5 mL of methanol/water (80:20, v/v). Finally, the fraction containing *Aloe* sterols was eluted from the cartridge with 10 mL of methanol. The volume of the eluate was adjusted to 20 mL with methanol and filtered through a 0.22 μm filter to obtain a measurement sample.

### AVGE capsules

Although commercially unavailable, AGVE capsules were manufactured for the use as food containing Aloe sterol. The capsules contain hydroxypropyl methylcellulose as the main component, with 0.25 g of AVGE powder and excipients per one capsule. The AVGE powder is composed of AVGE oil, starch degradation product, and glycerin fatty acid ester.

To pre-treat the AVGE capsules, two capsules were weighed and added to a 100 mL beaker, together with 40 mL of a 0.9% sodium chloride solution. After stirring for 30 min at room temperature and when the capsules had dissolved, the volume was adjusted to 50 mL with the 0.9% sodium chloride solution. This sample solution (5 mL), the 0.9% sodium chloride solution (5 mL), and the extraction solvent (10 mL) were added to a 50 mL tube made of polymethylpentene and shaken (approximately 320 rpm, room temperature, 10 min). After centrifugation (1,150 × *g*, room temperature, 10 min), the supernatant was collected, the extraction solvent (10 mL) was added to the precipitate, and the mixture was shaken and centrifuged under the same conditions as before, to collect the supernatant. The solvent was removed from the recovered supernatant by evaporation under a nitrogen stream at 40 °C. The dried residue was dissolved in 4 mL of methanol by sonication at room temperature for 10 min, 1 mL of distilled water was added, and the mixture was thoroughly mixed and filtered through a 0.22 μm filter. After that, the cleanup of the extract was performed using the previously described method for preparing the AVGE oil measurement sample.

Stability evaluation of *Aloe* sterol concentrations in AVGE capsules

The concentration of *Aloe* sterol in AVGE capsules used as a test food (test product) in a clinical trial [8] was measured before and 1 year after starting the clinical trial period.

The five compounds of *Aloe* sterol in the test food used in the clinical trial were divided into the following two groups: compounds with a common basic structure, that is, lophenol compounds (lophenol, 24-methyl-lophenol, and 24-ethyl-lophenol), and cycloartanol compounds (cycloartenol and 24-methylene-cycloartanol). These two compound groups were set as the standard and their quantitative values were calculated, and the results were obtained.” You may also consider deleting the sentence.

## Results and discussion

### Linearity

The coefficient of determination of the calibration curve (10 to 160 ng/mL) of the standard solution was higher than 0.999, which indicated that the fitting was acceptable.

Calculation of the limit of quantification and relative standard deviation (RSD)

The standard deviation, limit of quantification (10 S/N), and RSD results are shown in Table 2. Since the RSD was 5.1%, 3.4%, 2.6%, 4.4%, and 4.2% (*n* = 5) for lophenol, 24-methyl-lophenol, 24-ethyl-lophenol, cycloartanol, and 24-methylene-cycloartanol, respectively, very good repeatability was achieved. The limit of quantification (LOQ) was 4.0, 3.4, 2.3, 2.5, and 4.1 ng/mL for lophenol, 24-methyl-lophenol, 24-ethyl-lophenol, cycloartanol, and 24-methylene-cycloartanol, respectively.

**Table 2:** Calculation of limit of quantification and RSD (standard solution (10 ng/ml): *n* = 5).

compound	RSD (%)	limit of quantification (ng / mL)	SD
lophenol	5.1	4.0	0.54
24-methyl-lophenol	3.4	3.4	0.35
24-ethyl-lophenol	2.6	2.3	0.25
cycloartanol	4.4	2.5	0.45
24-methylene-cycloartanol	4.2	4.1	0.41

### Recovery test

The recovery test results are shown in Table 3: 95, 99, 103, 103, and 105% for lophenol, 24-methyl-lophenol, 24-ethyl-lophenol, cycloartanol, and 24-methylene-cycloartanol, respectively, indicating high-level recovery.

**Table 3:** Recovery test (*Aloe vera* gel powder: *n* = 3).

compound	Spiked level (ng / mL)	RSD (%)	Recovery (%)
lophenol	20	1.3	95
24-methyl-lophenol	20	2.5	99
24-ethyl-lophenol	20	2.2	103
cycloartanol	20	2.1	103
24-methylene-cycloartanol	20	1.4	105

### Precision

As shown in Table 4, this method enabled quantification of *Aloe* sterols with high-level precision.

**Table 4:** Precision (*Aloe vera* gel powder, measured value (μg/g) ± RSD (%)).

compound	Repeatability (*n* = 4)	Intermediate precision (*n* = 12)
lophenol	14.2±3.0	12.9±7.3
24-methyl-lophenol	22.2±3.1	21.2±6.8
24-ethyl-lophenol	9.6±6.4	8.9±6.5
cycloartanol	31.6±2.6	32.4±3.8
24-methylene-cycloartanol	33.2±2.3	33.2±5.2

Extraction efficiency of each *Aloe* sterol component by supercritical carbon dioxide

The *Aloe* sterols present in the raw material AVGP and AVGE were quantitated and their content ratios calculated. As shown in Table 5, the content ratio of the five sterols was 9%, 20%, 11%, 32%, and 28% for AVGP and 11%, 20%, 10%, 30%, and 29% for AVGE for lophenol, 24-methyl-lophenol, 24-ethyl-lophenol, cycloartanol, and 24-methylene-cycloartanol, respectively. Therefore, it was considered that the extraction efficiency of each *Aloe* sterol of AVGP by supercritical extraction was almost equivalent.

**Table 5:** Extraction efficiency of each *Aloe* sterol component by supercritical carbon dioxide (%).

compound	AVGP	AVGE
lophenol	9	11
24-methyl-lophenol	20	20
24-ethyl-lophenol	11	10
cycloartanol	32	30
24-methylene-cycloartanol	28	29

Stability of *Aloe* sterol concentrations in AVGE capsules used as test food in a clinical study

As shown in Table 6, the lophenol and cycloartanol compound concentrations were 9.3 μg/2 capsules and 11.6 μg/2 capsules, respectively, before the start of the study, and 9.1 μg/2 capsules and 11.7 μg/2 capsules after 1 year. Therefore, the concentration of *Aloe* sterol during the test period (12 weeks) was guaranteed.

**Table 6:** Stability of the amount of *Aloe* sterols in AVGE capsules during the clinical trial period (AVGE capsules, measured value (μg/2 capsules)).

Compound	Before the clinical trial period.	One year after starting the clinical trial period.
lophenol compounds	9.3	9.1
cycloartanol compounds	11.6	11.7

In this study, we developed a method to measure *Aloe* sterol from food ingredients (AVGP, AVGE oil, AVGE capsules). By measuring with LC-MS/MS, preprocessing was simple; as such, we believe that this is a high-throughput measurement method. In addition, validation using AVGP as a sample confirmed high recovery rate and accuracy. Moreover, the detection limit is very low. The content of *Aloe* sterol is 80 μg [14] per 1 g of AVGP and 20 μg [15] per 1 mg of AVGE, confirming aloe sterol as a trace component. There have been no reports measuring *Aloe* sterol by GC-MS; however, the quantification limit for β-sitosterol, a plant sterol with a similar structure, was reported as 0.05 μg/mL [16]. Since the content of *Aloe* sterol is less than one-tenth that of β-sitosterol, it was considered difficult to measure *Aloe* sterol by the method using GC-MS. On the contrary, the quantification limit of the developed method is 2.3 to 4.1 ng/mL, which enables accurate *Aloe* sterol measurement from food raw materials.

From previous studies, *Aloe* sterol has been found to be a functional compound. In order to develop functional foods containing *Aloe* sterol, it is essential to have a method that can reliably measure functional ingredients. In this test, as one of the applications of foods containing *Aloe* sterol, AVGE capsules were manufactured, and it was confirmed that there was no change in the amount of *Aloe* sterol due to storage for 1 year. We believe that these results will be extremely important and useful in developing functional foods containing *Aloe* sterol in the future.

## Declaration of Competing Interest

The authors declare that they have no known competing financial interests or personal relationships that could have appeared to influence the work reported in this paper.
